# Andrographolide Inhibits Mechanical and Thermal Hyperalgesia in a Rat Model of HIV-Induced Neuropathic Pain

**DOI:** 10.3389/fphar.2018.00593

**Published:** 2018-06-11

**Authors:** Zhihua Yi, Shuai Ouyang, Congfa Zhou, Lihui Xie, Zhi Fang, Huilong Yuan, Jinpu Yang, Lifang Zou, Tianyu Jia, Shanhong Zhao, Lin Li, Liran Shi, Yun Gao, Guilin Li, Shuangmei Liu, Hong Xu, Changshui Xu, Chunping Zhang, Shangdong Liang

**Affiliations:** ^1^Department of Physiology, Medical College of Nanchang University, Nanchang, China; ^2^Jiangxi Provincial Key Laboratory of Autonomic Nervous Function and Disease, Nanchang, China; ^3^Nursing College, Medical College of Nanchang University, Nanchang, China; ^4^School of Life Sciences, Nanchang University, Nanchang, China; ^5^Undergraduate Student of the Clinical Department, Medical College of Nanchang University, Nanchang, China; ^6^Department of Anatomy, Medical College of Nanchang University, Nanchang, China; ^7^Undergraduate Student of the Queen Mary School, Medical College of Nanchang University, Nanchang, China; ^8^Department of Cell Biology, Medical College of Nanchang University, Nanchang, China

**Keywords:** HIV gp120-associated neuropathic pain, anti-retroviral therapy-associated neuropathic pain, P2X7 receptor, dorsal root ganglia, andrographolide

## Abstract

**Aim:** In this study, we investigated whether andrographolide (Andro) can alleviate neuropathic pain induced by HIV gp120 plus ddC treatment and the mechanism of its action.

**Methods:** The paw withdrawal threshold and the paw withdrawal latency were observed to assess pain behaviors in all groups of the rats, including control group, control combined with Andro treatment group, sham group, gp120 combined with ddC treatment group, gp120 plus ddC combined with A438079 treatment group, and gp120 plus ddC combined with Andro treatment by intrathecally injecting at a dose of 25 μg/20 μl group. The protein expression levels of the P2X7 receptor, tumor necrosis factor-α-receptor (TNFα-R), interleukin-1β (IL-1β), IL-10, phospho-extracellular regulated protein kinases (ERK) (p-ERK) in the L4–L6 dorsal root ganglia (DRG) were measured by western blotting. Real-time quantitative polymerase chain reaction was used to test the mRNA expression level of the P2X7 receptor. Double-labeling immunofluorescence was used to identify the co-localization of the P2X7 receptor with glial fibrillary acidic protein (GFAP) in DRG. Molecular docking was performed to identify whether the Andro interacted perfectly with the rat P2X7 (rP2X7) receptor.

**Results:** Andro attenuated the mechanical and thermal hyperalgesia in gp120+ddC-treated rats and down-regulated the P2X7 receptor mRNA and protein expression in the L4–L6 DRGs of gp120+ddC-treated rats. Additionally, Andro simultaneously decreased the expression of TNFα-R and IL-1β protein, increased the expression of IL-10 protein in L4–L6 DRGs, and inhibited the activation of ERK signaling pathways. Moreover, Andro decreased the co-expression of GFAP and the P2X7 receptor in the SGCs of L4–L6 DRG on 14th day after surgery.

**Conclusion:** Andro decreased the hyperalgesia induced by gp120 plus ddC.

## Introduction

In 2015, an estimated 36.7 million HIV/AIDS cases reported worldwide, according to statistical data (WHO^1^). HIV infection and its associated treatments may cause a variety of peripheral neuropathies (Simpson, 2002; Ferrari et al., 2006; Ellis et al., 2010). The prevalence of peripheral neuropathies is as high as 69.4% in HIV-positive patients (Ghosh et al., 2012), and neuropathic pain is the major complaint of patients living with distal symmetric polyneuropathy (DSP). The precise pathogenesis of HIV-associated neuropathies remains unclear. Painful HIV-DSP may be induced by the envelope glycoprotein gp120 and other proteins (Yuan et al., 2014; Shi et al., 2016), as well as antiretroviral drug-induced neuropathy, including the use of nucleoside reverse transcriptase inhibitors (NRTIs), particularly dideoxynucleosides, zalcitabine (ddC), didanosine (ddI), and stavudine (d4T) (Verma et al., 2005). The combination of HIV-associated pathology and neurotoxic effects of anti-retroviral drugs is synergistic for the development of painful neuropathies (Wallace et al., 2007).

Dorsal root ganglia (DRG) play an important role in the pain signal transmitted from the peripheral nervous system to the central nervous system (Takeda et al., 2007). Adenosine triphosphate (ATP) is a critical molecule in the development and maintenance of chronic pain (Chizh and Illes, 2001; Burnstock, 2013, 2014, 2017). In DRG, satellite glial cells (SGCs) are the most abundant cell type enwrapping the neuronal soma (Hanani, 2005; Costa and Moreira Neto, 2015). ATP serves as a signaling molecule that modulates communication between neurons and glial cells (Hanani, 2005; Fields and Burnstock, 2006; Verderio and Matteoli, 2011; Lohr et al., 2014). Purinergic receptors include P1 and P2 receptors. The P2X7 receptor is expressed in DRG SGCs (Kobayashi et al., 2013). The P2X7 receptor modulates behavioral responses to painful stimuli (McGaraughty et al., 2007; Fulgenzi et al., 2008). Therefore, the specific blockage of the P2X7 receptor can prevent the deterioration of neuropathic pain (Jarvis, 2010; Gum et al., 2012).

As a natural medicine, andrographolide (Andro) has antibacterial, anti-asthmatic, antiviral, neuroprotective, and anti-inflammatory effects (Wiart et al., 2005; Abu-Ghefreh et al., 2009; Chan et al., 2010; Li et al., 2015; Banerjee et al., 2017; Panraksa et al., 2017; Tan et al., 2017; Zhao et al., 2017). The derivative of Andro decreased histopathological changes, the recruitment of immune-inflammatory cells, and the secretion of pro-inflammatory cytokines to produce anti-inflammatory effects (Liu et al., 2014; Yang et al., 2016). It was reported that Andro improved diabetes and ameliorated diabetic-related complications through reducing the expression of pro-inflammatory cytokines (Islam, 2017). Andro suppressed LPS-induced microglial MIP-1α, P2X7 receptor, and its downstream signaling mediators including inflammasome NLRP3, caspase1, and mature IL-1β (Das et al., 2017). To date, the effect of Andro on the P2X7 receptor-mediated neuropathic pain has not been investigated. In this study, we determined the therapeutic effects and probable mechanisms of Andro in peripheral neuropathic pain induced by gp120 associated with ddC in rats.

## Materials and Methods

### Animals and Surgical Methods

Healthy male Sprague-Dawley (SD) rats with weights of 200–250 g were used in this study, and they were provided by the Center of Laboratory Animal Science of Nanchang University. There were four rats per cage, which was kept clean, food and water were easily obtained, and the room was maintained on a 12:12 h light:dark schedule with a room temperature of 21°C, and 60% humidity. The use of these animals was reviewed and approved by the Animal Care and Use Committee of the Medical College of Nanchang University. All experiments were implemented according to the Guidelines of the US National Institutes of Health (NIH) regarding the care and use of animals for experimental procedures. The rats were randomly arranged into different groups as follows (with eight rats in each group): the control group; control+andro group; the HIV-gp120 plus 2′,3′-dideoxycytidine (ddC) (intraperitoneal injection, i.p.) group (gp120+ddC group); the HIV-gp120 plus ddC rats treated with the Andro group (gp120+ddC+andro group); gp120+ddC+A438079 (P2X7 antagonist, AdooQ BioScience) group; and the sham operation group (sham group).

A previously described technique was used for the perineural HIV-gp120 administration (Yi et al., 2017). Briefly, under 10% chloral hydrate anesthesia (3 ml/kg, i.p., supplemented as necessary), the left sciatic nerve of the SD rats was exposed in the popliteal fossa, without damaging the nerve construction. A 2 × 6 mm strip of oxidized regenerated cellulose was previously soaked in 250 μl of a 0.1% rat serum albumin (RSA) saline solution containing 400 ng of gp120 (Sigma, St. Louis, MO, United States) or 0.1% RSA in saline for the sham surgery. A 2 × 6 mm length of the sciatic nerve proximal to the trifurcation was wrapped loosely with the soaked cellulose, which did not cause any nerve constriction, and was left *in situ*. The incision was closed with 4/0 sutures (Yi et al., 2017). Then, the animals received an i.p. of ddC (one of NRTIs, 20 mg/kg, Sigma, St. Louis, MO, United States) after surgery. The ddC was freshly prepared in saline on the day of the experiment, the rats in the ddC group were administered an i.p. injection of ddC (0.5 ml, 20 mg/kg), followed by a further ddC i.p. frequency of three times a week with the same dose. The animals in the sham group received the same volume of saline injection. The animals were randomly assigned to experimental groups, excluding those with motor deficits. Andro (QingFeng Pharmaceutical Co., Ltd., Jiangxi, China) was intrathecally (i.t.) injected into the rats in the gp120+ddC+andro group every other day from day 1 post-surgery until to the 14th day after surgery, and the i.t. injections were performed under anesthesia with diethyl ether at a dose of 25 μg (a volume of 20 μl). A438079 treatment in the gp120+ddC group was i.t. injected into rats once on 7th day after surgery at the concentration of 10 μM (the volume of 20 μl).

To prove the analgesic effect of Andro through reduction of P2X7 receptor activity, supplementary experiments were made. The rats were randomly arranged into the following groups: sham group, gp120+ddC group, gp120+ddC+andro group, gp120+ddC+A438079 group, and gp120+ddC+andro+A438079 group (eight rats for each group). On the 7th day, Andro and A438079 were i.t. injected into the corresponding group of rats, respectively. The doses were the same as before.

### Measurement of the Paw Withdrawal Threshold (PWT)

Measurements of the paw withdrawal threshold (PWT) were obtained on day 0 (before operation), as well as on days 1, 3, 5, 7, 9, 11, and 14 after operation. The determination of the PWT was performed between the hours of 8:00 and 12:00 by using a BME-404 electronic mechanical stimulator (Institute of Biomedical Engineering, Chinese Academy of Medical Sciences, Tianjin, China). The main technical parameters of this equipment were as follows: end face diameter of test needle, 0.6 mm; pressure measurement range, 0.1–50 g; and a pressure measurement resolution, 0.05 g, the pressure value was automatically recorded once the needle touches the hind paw of the rat. An organic glass box (22 × 22 × 12 cm) was placed on the sieve of the metal frame. The rats were placed into the box for a 30-min adaptation. The left hind paws were touched with the test needle until the escaping behavior appeared. An effective escaping behavior response was defined as an immediate withdrawal and/or licking of the paw to application of the stimulus. An effective response to a stimulus was followed by the next smaller power, while an invalid response was followed by the next bigger power. Measurements were performed two to three times for each rat (interval, ≥5 min), and the mean value was calculated as PWT for this measurement (Lin et al., 2010; Zhang et al., 2013; Nasirinezhad et al., 2015; Yi et al., 2017). Two-way ANOVA was performed and followed by a Tukey’s HSD *post hoc* test (SPSS 22.0, SPSS Inc.) for multiple testing.

### Measurement of the Paw Withdrawal Latency (PWL)

Measurements of the paw withdrawal latency (PWL) were also obtained on day 0 (before operation), as well as on days 1, 3, 5, 7, 9, 11, and 14 after operation. The Thermal Paw Stimulation System (BME-410C, Tianjin, China) was used to measure the latency of hind paw withdrawal to a thermal stimulus. The rats were placed in a transparent, square bottomless acrylic box (22 cm × 12 cm × 22 cm) on a glass plate with a light located underneath, and the plantar surface of the hind paw of each rat was exposed to the radiant heat. A beam of radiant heat was applied through the glass floor to the plantar surface of the hind paw after a 30-min habituation period. Activation of the bulb simultaneously activated a timer, and both the bulb and the timer were immediately turned off by paw withdrawal or at the 30-s cut-off time. A blinded observer tested the hind paws two to three times at 5-min intervals (Lin et al., 2010; Zhang et al., 2013; Nasirinezhad et al., 2015; Yi et al., 2017). Two-way ANOVA was performed and followed by a Tukey’s HSD *post hoc* test (SPSS 22.0, SPSS Inc.) for multiple testing.

### RNA Extraction and Real-Time (RT) Quantitative Polymerase Chain Reaction (qPCR)

The rats in all groups were anesthetized by 10% chloral hydrate (3 ml/kg, i.p.) on the 14th day after the operation. The L4–6 DRGs were immediately isolated and flushed with ice-cold phosphate-buffered saline (PBS). The total RNA samples were prepared from the L4–L6 DRGs of each group using the TRIzol Total RNA Reagent (Beijing Tiangen Biotech Co.). The cDNA synthesis was performed with 2 μg of total RNA using the RevertAid First Strand cDNA Synthesis Kit (Thermo Fisher Scientific, United States). The primers were designed with Primer Express 3.0 software (Applied Biosystems, Inc., Foster City, CA, United States) and the following sequences: P2X7, forward 5′-GAGTCCGAGGCAATCTAATG-3′; reverse 5′-CTGTGATCCCAACAAAGGTC-3′; and β-actin, forward 5′-TAAAGACCTCTATGCCAACACAGT-3′, reverse 5′-CACGATGGAGGGGCCGGACTCATC-3′. Quantitative PCR was performed using the SYBR^®^ Green Master Mix in an ABI PRISM^®^ 7500 Sequence Detection System (Applied Biosystems, Inc., Foster City, CA, United States). The quantification of gene expression was performed using the ΔΔCT calculation with CT as the threshold cycle. The relative levels of target genes, normalized to the sample with the lowest CT, were given as 2^-ΔΔCT^ (Tu et al., 2013). The β-actin was used to be internal control in the groups. The relative expression levels of mRNA in the all groups were normalized to β-actin.

### Western Blotting

On the 14th day after the operation, the animals were anesthetized, and tissue collection was performed as described above, except that the tissues were snap-frozen in tubes on dry ice during the collection (Lin et al., 2010; Yi et al., 2017). Briefly, the animals were anesthetized with chloral hydrate, and the L4–6 DRGs were dissected. The DRGs were immediately isolated and rinsed in ice-cold PBS. The ganglia were homogenized by mechanical disruption in lysis buffer containing the following: 50 mM of Tris–HCl, pH 8.0, 150 mM of NaCl, 0.1% sodium dodecyl sulfate (SDS), 1% Nonidet P-40, 0.02% sodium deoxycholate, 100 μg/ml of phenylmethylsulfonyl fluoride, and 1 μg/ml of Aprotinin. The cells were incubated on ice for 50 min. The homogenates were subsequently centrifuged at 12,000 rpm for 10 min, and the supernatants were collected. The quantity of total proteins in the supernatants was determined using the Lowry method. After dilution with loading buffer (250 mM of Tris–HCl, 200 mM of dithiothreitol, 10% SDS, 0.5% bromophenol blue, and 50% glycerol) and heating to 95°C for 5 min, samples containing equal amounts of protein (20 μg) were separated by 10% SDS–polyacrylamide gel electrophoresis using a Bio-Rad system. The proteins were subsequently transferred onto PVDF membranes by electrophoretic transfer using the same system. The membrane was blocked with 5% non-fat dry milk in 25 mM of Tris-buffered saline, pH 7.2, plus 0.05% Tween 20 (TBST) for 2 h at room temperature, followed by incubation with a rabbit anti-P2X7 receptor (1:200, Alomone Lab, Israel), or with other antibodies such as rabbit anti-tumor necrosis factor-α-receptor (TNF-α-R), rabbit anti-interleukin-1β (IL-1β) and mouse anti-IL-10 (1:500 dilutions, Abcam, Co.), anti-total extracellular regulated protein kinases (ERK)1/2 and anti-phospho-ERK1/2 MAPK (1:1000, Cell Signaling Technology), and mouse monoclonal anti-β-actin antibody (1:800 dilutions, Beijing Zhongshan Biotech Co., China) at 4°C overnight. The membranes were washed three times with TBST and incubated (1 h, room temperature) with a horseradish peroxidase-conjugated secondary antibody (goat anti-mouse or goat anti-rabbit IgG; 1:2000; Beijing Zhongshan Biotech Co., China) in blocking buffer. After another wash cycle, the labeled proteins were visualized by enhanced chemiluminescence (ECL, Thermo Fisher Scientific, United States) on high-performance film (Shanghai Pufei Biotech Co.). The chemiluminescent signals were collected on autoradiography film, and the band intensity was quantified using Image-Pro Plus software. The relative band intensity of the target proteins was normalized against the intensity of the respective β-actin internal control.

### Double-Labeling Immunofluorescence

On the 14th day after the operation, the L4–L6 DRG was dissected and fixed in 4% paraformaldehyde (PFA) diluted in PBS [145 mM of NaCl, 7.3 mM of Na_2_HPO_4_, and 2.7 mM of NaH_2_PO_4_ (pH 7.2)] for 24 h. The ganglia were cryopreserved in 20% sucrose overnight at 4°C and then cut into 8-μm-thick sections under the temperature of -20°C. The sections were rinsed three times for 5 min each in PBS, followed by 0.1% Triton X-100 in PBS for 30 min at room temperature, and nonspecific staining was blocked by incubation with 10% normal goat serum (Jackson ImmunoResearch, Inc., West Grove, PA, United States). Cell nuclei were revealed by the 4′,6-diamidino-2-phenylindole [dihydrochloride (DAPI), Thermo Fisher Scientific, United States, Catlog Number: D1306] fluorescent staining in this study. The sections were then incubated with rabbit anti-P2X7 receptor (1:50, Alomone Lab, Israel), combined with mouse anti-glial fibrillary acidic protein (GFAP) (1:100, Abcam, Co.) overnight at 4°C. The sections were subsequently incubated with goat anti-rabbit (1:200, TRITC, EarthOx, United States) and goat anti-mouse IgG (1:200, FITC, EarthOx, United States) for 1 h at 37°C. After the development of the DAPI fluorescent staining for 2 min, the slides were washed with distilled water (Xie et al., 2017). Finally, the sections were washed once more in PBS, and results were assessed using a 20× objective fluorescence microscope (Olympus, Tokyo, Japan). Data were collected from eight animals in each group. Five fields containing approximately 50 neurons were randomly selected, and data analysis from each animal was averaged. Image-Pro Plus 6.0 image analysis software (Media Cybernetics, Inc.) was used to quantify the expression levels of the P2X7 receptor, and GFAP. Briefly, the fluorescence intensity value of each group of fluorescent pictures was collected and calculated, then the fluorescence intensity value was multiplied by the corresponding area of each group of fluorescent pictures, thus got the final value of fluorescent intensity. Experiments were implemented for eight times. Bar graphs show the statistic fluorescence intensity value of each group, Student’s *t*-test was performed to compare two groups’ of significance. To specify the immunoreactivity of the P2X7 receptor, and GFAP, normal goat serum and PBS were used as negative controls instead of the primary antibodies. In DRG, each neuron is surrounded by its own glial cover, and together they form a distinct unit, largely isolated from other similar units in the ganglion (Hanani, 2005; Dublin and Hanani, 2007; Feldman-Goriachnik and Hanani, 2017). GFAP is a marker of activated SGCs. The cells surrounded by GFAP in the images should be neurons. And neurons that were surrounded by GFAP-positive SGCs by >50% of their circumference were counted and expressed as a percentage of the total number of neurons present in the fields analyzed. Histogram showed that the number of neurons surrounded with GFAP and P2X7 positive SGCs in the SCGs.

### Molecular Docking

Molecular docking is a computer simulation tool that attempts to predict the binding mode of a ligand in the active site of a protein. Molecular docking studies mimic the natural interaction of a ligand with the protein. We performed molecular docking computations by using AutoDock 4.2 (Morris et al., 1996; Trott and Olson, 2010; Rao et al., 2017). The technique of docking is to position the ligand in different orientations and conformations within the binding site to calculate optimal binding geometries and energies. Therefore, after the docking procedure, the proper conformation of ligand in the active site of protein is obtained and used for calculation of molecular descriptors. For each ligand, a number of configurations, called poses, were generated and scored (Trott and Olson, 2010; Rao et al., 2017). The score can be calculated as either a free energy of binding, which considers salvation and entropy, the enthalpic term of the free energy of binding, or a qualitative shaped-based numerical measure. The final top-scoring poses, along with their scores and conformation energies, are written to a database for further analysis.

According to rat P2RX_7_ (rP2RX_7_) protein sequence NP_062129 as a target protein, the structure of the P2RX_7_ protein 3D file was obtained by homology modeling via on line SWISS-MODEL website^2^. Andro, PubChem CID 5318517 was used as a ligand. Both ligands were prepared with AutoDockTools (ADT) and Python scripts, namely, prepare_ligand4.py and prepare_receptor4.py, in the AutoDock4.2 program. The binding pocket position in target protein was specified with the ADT molecular viewer. The parameters were maintained at their default values. Finally, the output files were viewed by using MGLtools and PyMol^3^. The chemical structural formula of Andro from https://pubchem.ncbi.nlm.nih.gov/compound/531 8517#section=Top was used.

### Statistical Analysis

The data were analyzed using SPSS 22.0 software. The numerical values are reported as the mean ± SD. Statistical significance was determined by Student’s *t*-test for two group comparisons, and one-way analysis of variance (ANOVA) followed by a Tukey’s HSD *post hoc* test (SPSS 22.0, SPSS Inc.) for simple testing, and two-way ANOVA followed by a Tukey’s HSD *post hoc* test (SPSS 22.0, SPSS Inc.) for multiple comparisons. A value of *p* < 0.05 was considered to be statistically significant.

## Results

### Andrographolide Downregulated the High Expression of the P2X7 Receptor in Gp120 Plus ddC-Treated Rats

**FIGURE 1:**
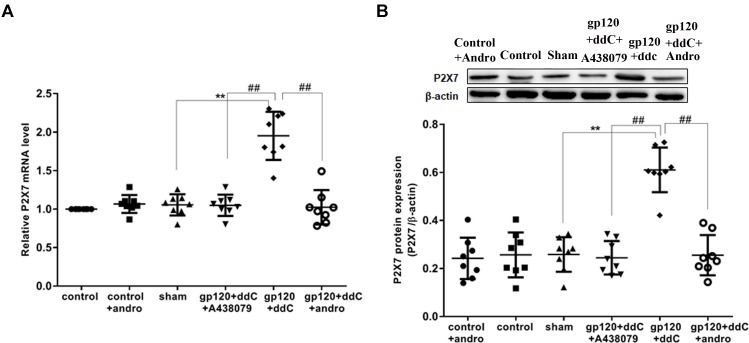
Effects of the Andro treatment on the expression of P2X7 mRNA and protein in L4–6 DRGs from each group. **(A)** Expression of P2X7 mRNA in the DRG was measured by RT-qPCR (*n* = 8 per group). Expression in the gp120+ddC group was higher than that in the sham group (*p* < 0.01). In gp120+ddC+andro rats, the expression was significantly lower than that in the gp120+ddC rats (*p* < 0.01), which was same as the gp120+ddC+A438079-treated rats. No significant difference was found between the sham group and the control group or the control+andro group. Data are presented as the means ± SE. ^∗∗^*p* < 0.01 compared to the sham group; ##*p* < 0.01 compared to the gp120+ddC-treated group. **(B)** Expression of P2X7 protein in the DRG was assessed by western blotting (*n* = 8 for each group). P2X7 protein expression in the gp120+ddC-treated group was increased compared to the sham group (*p* < 0.01). In gp120+ddC+andro rats, the expression was significantly lower than that in the gp120+ddC rats (*p* < 0.01), which was same as the gp120+ddC+A438079-treated rats. No significant difference was found between the sham group and the control group or the control+andro group. Bar graphs show the ratio of the P2X7 receptor protein level to β-actin level in each group. Data are displayed as the means ± SE. ^∗∗^*p* < 0.01 compared to the sham group; ##*p* < 0.01 compared to the gp120+ddC group.

The expression of the P2X7 mRNA in the DRG was measured by RT-qPCR. There was no significant difference in the relative levels of the P2X7 mRNA between the sham group and the control group or the control+andro group (*p* > 0.05). The studies showed that the expression levels of the P2X7 mRNA in the gp120+ddC group were significantly increased compared to those in the sham group (*p* < 0.01, *n* = 8 for each group). The relative levels of the P2X7 mRNA in the gp120+ddC+andro group rats were significantly reduced compared to those in the gp120+ddC rats (*p* < 0.01), which were in consistent of the P2X7 receptor antagonist A438079 treatment group (**Figure 1A**).

The expression levels of the P2X7 protein in the DRG were analyzed by western blotting. There was no significant difference in the relative levels of P2X7 protein between the sham group and the control group or the control+andro group (*p* > 0.05). Using image analysis, the P2X7 protein expression (normalized to each β-actin internal control) in the gp120+ddC group was significantly enhanced compared to that in the sham group (*p* < 0.01, *n* = 8 for each group). The relative levels of P2X7 protein expression in the gp120+ddC+andro group were much lower than those in the gp120+ddC group (*p* < 0.01), which were in consistent of the P2X7 receptor antagonist A438079 treatment group (**Figure 1B**). The results indicated that gp120 combined with ddC treatment upregulated the expression of P2X7 receptor mRNA and protein, whereas intrathecal treatment with Andro decreased the upregulated expression of P2X7 mRNA and protein in the gp120+ddC treatment group.

### Reduction of the P2X7 Receptor by Andrographolide Was Involved in Relieving Hyperalgesia of Gp120 Plus ddC-Treated Rats

**FIGURE 2:**
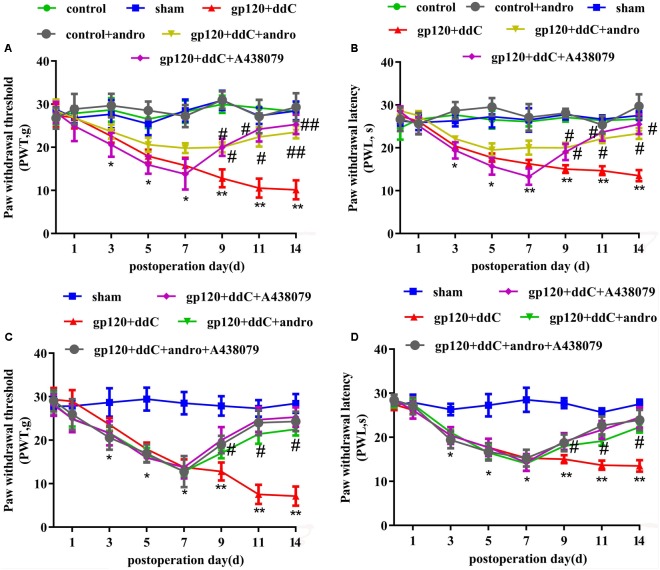
Treatment with Andro relieves mechanical and thermal hyperalgesia in gp120+ddC-treated rats. **(A)** The PWT in the gp120+ddC group was lower than that in the sham group (*p* < 0.01). No significant difference was found between the sham group, control+andro group and the control group (*p* > 0.05). In gp120+ddC+andro rats, the PWT was gradually higher than that in the gp120+ddC group (*p* < 0.01), which was same as the gp120+ddC+A438079-treated rats. Each group consisted of eight rats (*n* = 8 for each group). Data are displayed as the means ± SE. ^∗^*p* < 0.05 compared to the sham group; ^∗∗^*p* < 0.01 compared to the sham group; #*p* < 0.05 compared to the gp120+ddC-treated group; ##*p* < 0.01 compared to the gp120+ddC-treated group (two-way ANOVA test, *F*_(7,336)_ = 35.46 for comparisons from different post-operation days, *F*_(5,336)_ = 216.4 for comparisons from different groups of rats). **(B)** The PWL in the gp120+ddC group was lower than that in the sham group (*p* < 0.01). No significant difference was found between the sham group, control+andro group and the control group (*p* > 0.05). In gp120+ddC+andro rats, the PWL became gradually higher than that in the gp120+ddC group (*p* < 0.05), which was same as the gp120+ddC+A438079-treated rats. Each group consisted of eight rats (*n* = 8 for each group). Data are displayed as the means ± SE. ^∗^*p* < 0.05 compared to the sham group; ^∗∗^*p* < 0.01 compared to the sham group; #*p* < 0.05 compared to the gp120+ddC-treated group (two-way ANOVA test, *F*_(7,336)_ = 56.81 for comparisons from different post-operation days, *F*_(5,336)_ = 274 for comparisons from different groups of rats). **(C)** The PWT in gp120+ddC group rats was lower than that in the sham group rats (*p* < 0.01), but there was no significant deference for the PWT between the gp120+ddC+andro group and gp120+ddC+andro+A438079 group (*p* > 0.05). The PWT in the gp120+ddC+andro group rats was higher than that in the gp120+ddC group rats (*p* < 0.01). Result in gp120+ddC+andro+A438079 group was the same as that in gp120+ddC+A438079 group (*p* > 0.05). Each group consisted of eight rats (*n* = 8 for each group). Data are displayed as the means ± SE. ^∗^*p* < 0.05 compared to the sham group; ^∗∗^*p* < 0.01 compared to the sham group; #*p* < 0.05 compared to the gp120+ddC-treated group (two-way ANOVA test, *F*_(7,200)_ = 87.79 for comparisons from different post-operation days, *F*_(4,200)_ = 131.8 for comparisons from different groups of rats). **(D)** The PWL in gp120+ddC group rats was lower than that in the sham group rats (*p* < 0.01), but there was no significant deference for the PWL between the gp120+ddC+andro group and the gp120+ddC+andro+A438079 group (*p* > 0.05). The PWL in the gp120+ddC+andro group rats was higher than that in the gp120+ddC group rats (*p* < 0.01). Result in gp120+ddC+andro+A438079 group was the same as that in gp120+ddC+A438079 group (*p* > 0.05). Each group consisted of eight rats (*n* = 8 for each group). Data are displayed as the means ± SE. ^∗^*p* < 0.05 compared to the sham group; ^∗∗^*p* < 0.01 compared to the sham group; #*p* < 0.05 compared to the gp120+ddC-treated group (two-way ANOVA test, *F*_(7,200)_ = 157.7 for comparisons from different post-operation days, *F*_(4,200)_ = 189.9 for comparisons from different groups of rats).

Mechanical hyperalgesia was tested with a mechanical stimulator (*n* = 8 for each group). There was no significant difference in the PWT between the sham group and the control group or the control+andro group (*p* > 0.05). At 5–14 days after the operation, the PWT in the gp120+ddC group became gradually lower than that in the sham group (*p* < 0.01). The PWT in the gp120+ddC+andro group became gradually higher than that in the gp120+ddC group from day 5 to day 14 (*p* < 0.05 and *p* < 0.01), which were in consistent of the P2X7 receptor antagonist A438079 treatment group (two-way ANOVA test, *F*_(7,336)_ = 35.46 for comparisons from different post-operation days, *F*_(5,336)_ = 216.4 for comparisons from different groups of rats) (**Figure 2A**).

Thermal hyperalgesia was tested with a Thermal Paw Stimulation System (*n* = 8 for each group). There was no significant difference in the PWL between the sham group and the control group or the control+andro group (*p* > 0.05). At 5–14 days after the operation, the PWL in the gp120+ddC group became gradually shorter than that in the sham group (*p* < 0.01). The PWL in the gp120+ddC+andro group became gradually longer than that in the gp120+ddC group from day 5 to day 14 (*p* < 0.05 and *p* < 0.01), which were in consistent of the P2X7 receptor antagonist A438079 treatment group (two-way ANOVA test, *F*_(7,336)_ = 56.81 for comparisons from different post-operation days, *F*_(5,336)_ = 274 for comparisons from different groups of rats) (**Figure 2B**).

In order to identify if analgesic effect of Andro was through reduction of P2X7 receptor activity, the occlusive role of A438079 in the analgesic effect of Andro was observed. The PWT and PWL were implemented on days 1, 3, 5, 7, 9, 11, and 14 after surgery. The PWT and PWL in gp120+ddC group rats were lower than those in the control group rats (*p* < 0.01), but there was no significant deference for the PWT and PWL between the gp120+ddC+andro group and gp120+ddC+andro+A438079 group (*p* > 0.05). The PWT and PWL in the gp120+ddC+andro group rats were higher than those in the gp120+ddC group rats (*p* < 0.01). Results in gp120+ddC+andro+A438079 group were the same as those in gp120+ddC+A438079 group (*p* > 0.05) (**Figures 2C,D**) (C: two-way ANOVA test, *F*_(7,200)_ = 87.79 for comparisons from different post-operation days, *F*_(4,200)_ = 131.8 for comparisons from different groups of rats; D: two-way ANOVA test, *F*_(7,200)_ = 157.7 for comparisons from different post-operation days, *F*_(4,200)_ = 189.9 for comparisons from different groups of rats). The results suggest that in the presence of P2X7 receptor antagonist, Andro no longer shows higher analgesic effects.

The results suggested that gp120 combined with ddC treatment induced mechanical hyperalgesia and thermal hyperalgesia. After intrathecal treatment with Andro, the hyperalgesia in the gp120+ddC group was attenuated. This finding indicated that Andro decreased the expression of P2X7 mRNA and protein in the gp120+ddC rats and relieved the hyperalgesia by downregulating the expression of the P2X7 receptor. In addition, the analgesic effect of Andro may also have reduction of P2X7 receptor activity.

### Reduction of the P2X7 Receptor by Andrographolide Lessened the Upregulated Co-expression Values of P2X7 and GFAP in the DRG of Gp120 Plus ddC-Treated Rats

**FIGURE 3:**
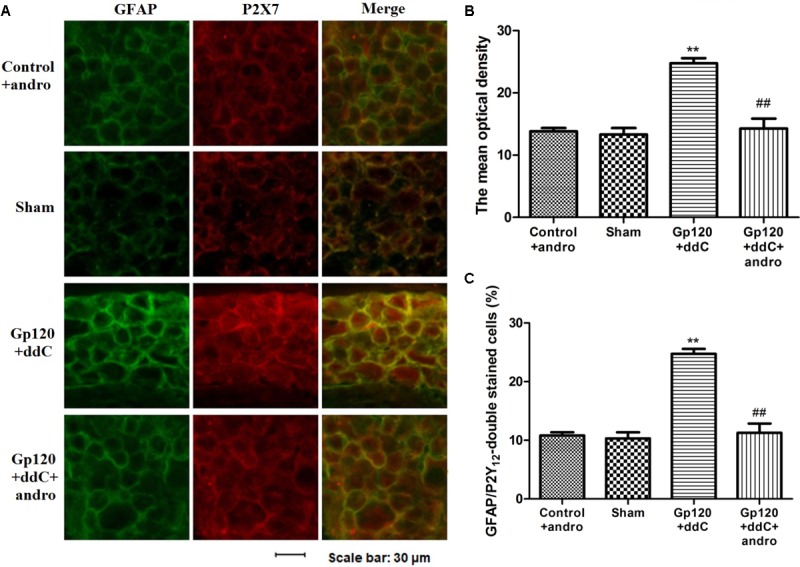
Treatment with Andro reduces the upregulated co-expression values of the P2X7 receptor and GFAP in the DRG of gp120+ddC-treated rats. **(A)** The co-expression of P2X7 receptor and GFAP was analyzed on the 18–20th day after surgery. Co-expression of the P2X7 receptor and GFAP in the gp120+ddC group was higher than that in the sham group. No difference was found between control+andro and sham rats. Treatment with Andro reduced the co-expression values of the P2X7 receptor and GFAP in gp120+ddC+andro-treated rats compared with gp120+ddC-treated rats that did not receive Andro treatment (*n* = 8 for each group). Scale bar: 30 μm. **(B)** Histogram showed that the IOD value for co-expression of the P2X7 receptor and GFAP in the DRG. **(C)** Histogram showed that the number of neurons surrounded with GFAP- and P2X7-positive SGCs in the DRG SCGs. The data are shown as the mean ± SD. ^∗∗^*p* < 0.01, compared to the sham group; ##*p* < 0.01, compared to the gp120+ddC group (*n* = 8 for each group).

As a marker of SGCs, the upregulation of GFAP in SGCs suggests the activation of SGCs. Co-expression values of P2X7 and GFAP in the DRG of gp120+ddC-treated rats were tested by double-labeling immunofluorescence. The co-expression values of P2X7 and GFAP in the gp120+ddC group were higher than those in the sham group. No difference was found between the control+andro and the sham rats, while after the treatment of Andro, the co-expression values of the P2X7 receptor and GFAP in gp120+ddC+andro-treated rats were lessened compared with those in gp120+ddC-treated rats (**Figures 3A,B**). The number of neurons surrounded with GFAP- and P2X7-positive SGCs in the DRG SCGs in the DRG after the gp120+ddC-treated rats was increased. Andro decreased the number of neurons surrounded with GFAP- and P2X7-positive SGCs in the DRG SCGs in the gp120+ddC-treated rats (**Figure 3C**). The results indicated that the P2X7 receptor in the SGCs was involved in the pathological injury of the gp120+ddC rats, and Andro decreased the co-expression values of P2X7 and GFAP in the activation of SGCs.

### Reduction of the P2X7 Receptor by Andrographolide Decreased the Expression of TNF-α-R and IL-1β Protein and Increased the Expression of IL-10 Protein in the DRG of Gp120 Plus ddC-Treated Rats

**FIGURE 4:**
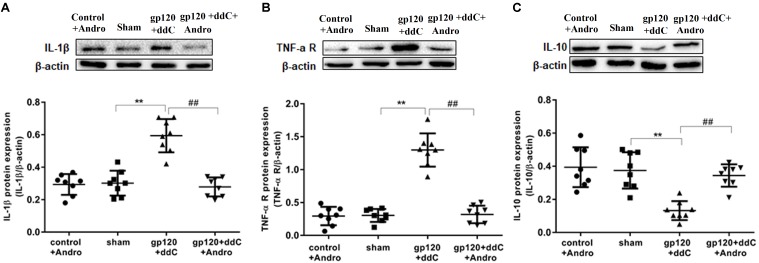
Effects of the Andro treatment on the expression of TNF-α-R, IL-1β, and IL-10 protein in L4–6 DRGs. **(A)** The expression of IL-1β protein in the DRG of the gp120+ddC group was increased compared to the sham group (*p* < 0.01). The expression of IL-1β protein in the gp120+ddC+andro group was decreased compared to the gp120+ddC group (*p* < 0.01). The bar graphs show the ratio of the IL-1β protein level to β-actin level in each group, respectively. The data are shown as the mean ± SD. *p* < 0.01, compared to the sham group; ##*p* < 0.01, compared to the gp120+ddC group (*n* = 8 for each group). **(B)** The expression of TNF-α-R protein in the DRG of the gp120+ddC group was increased compared to the sham group (*p* < 0.01). The expression of TNF-α-R protein in the gp120+ddC+andro group was decreased compared to the gp120+ddC group (*p* < 0.01). The bar graphs show the ratio of the TNF-α-R protein level to β-actin level in each group, respectively. The data are shown as the mean ± SD. *p* < 0.01, compared to the sham group; ##*p* < 0.01, compared to the gp120+ddC group (*n* = 8 for each group). **(C)** The expression of IL-10 protein in the DRG of the gp120+ddC group was decreased compared to the sham group (*p* < 0.01). The expression of IL-10 protein in the DRG of the gp120+ddC+andro group was increased compared to the gp120+ddC group (*p* < 0.01). The bar graphs show the ratio of the IL-10 protein level to β-actin level in each group, respectively. The data are shown as the mean ± SD. ^∗∗^*p* < 0.01, compared to the sham group; ##*p* < 0.01, compared to the gp120+ddC group (*n* = 8 for each group).

The expression levels of TNF-α-R, IL-1β, and IL-10 proteins in DRG were analyzed by western blotting. Using image analysis, the values for TNF-α-R protein expression (normalized to each β-actin internal control) in the gp120+ddC group were significantly augmented compared to those in the sham group (*p* < 0.01, *n* = 8 for each group). There was no significantly difference in TNF-α-R protein expression between the sham group and the control+andro group (*p* > 0.05). The relative levels of TNF-α-R protein expression in the gp120+ddC+andro group were lower than those in the gp120+ddC group (*p* < 0.01) (**Figure 4B**).

Using image analysis, the values for IL-1β protein expression (normalized to each β-actin internal control) in the gp120+ddC group were significantly augmented compared to those in the sham group (*p* < 0.01, *n* = 8 for each group). There was no significant difference in IL-1β protein expression between the sham group and the control+andro group (*p* > 0.05). The relative levels of 1L-1β protein expression in the gp120+ddC+andro group were lower than those in the gp120+ddC group (*p* < 0.01) (**Figure 4A**).

The values for IL-10 protein expression (normalized to each β-actin internal control) in the gp120+ddC group were significantly lower compared to those in the sham group (*p* < 0.01, *n* = 8 for each group). There was no significant difference in IL-10 protein expression between the sham group and the control+andro group (*p* > 0.05). The relative levels of IL-10 protein expression in the gp120+ddC+andro group were much higher than those in the gp120+ddC group (*p* < 0.01) (**Figure 4C**).

The results showed that Andro treatment decreased the release of pro-inflammatory cytokines and increased the release of anti-inflammatory cytokines (e.g., IL-10) in the gp120+ddC group.

### Reduction of the P2X7 Receptor by Andrographolide Inhibited the Activation of ERK Pathway in the DRG of Gp120+ddC-Treated Rats

**FIGURE 5:**
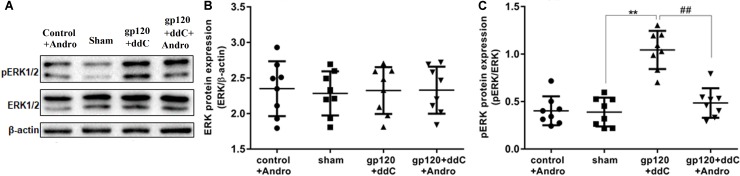
Effects of Andro treatment on the expression of ERK1/2 and p-ERK1/2 in L4–6 DRGs. **(A)** The expression levels of ERK1/2 and p-ERK1/2 in the DRG were analyzed using western blotting. **(B)** The IOD ratio of ERK1/2 to β-actin was not significantly different among the four groups: the gp120+ddC group, the sham group, the control+andro group, and the gp120+ddC+andro group (one-way ANOVA test, *F*_(3,28)_ = 0.05357, *p* = 0.9833 for comparisons from different groups). **(C)** The bar graphs show that the IOD ratio of p-ERK1/2 to ERK1/2 in the gp120+ddC group was higher than that in the sham group (*n* = 8) and the IOD ratio of p-ERK1/2 to ERK1/2 in the gp120+ddC+andro-treated rats was significantly lower than that in the gp120+ddC group (*p* < 0.01, *n* = 8 for each group). The data represent the mean ± SD, *n* = 8. ^∗∗^*p* < 0.01 compared to the sham group; ##*p* < 0.01 compared to the gp120+ddC group.

The phosphorylation and activation of ERK are involved in inflammatory pain. The expression levels of ERK and p-ERK in the DRG were analyzed by western blotting. The integrated optical density (IOD) ratio of ERK to β-actin was not significantly different between the gp120+ddC group and the sham group (*p* > 0.05). There was also no significant difference between the sham group and the control+andro group (*p* > 0.05). However, the IOD ratio of p-ERK to ERK was higher in the gp120+ddC group than that in the sham group (*p* < 0.01, *n* = 8 for each group) (**Figures 5A,B**). There was no significant difference in the IOD ratio of p-ERK to ERK between the sham group and control+andro group (*p* > 0.05). The results indicated that the role of ERK phosphorylation in the DRG is related to the P2X7 receptor-mediated hyperalgesia in the gp120+ddC-treated rats (one-way ANOVA test, *F*_(3,28)_ = 0.05357, *p* = 0.9833 for comparisons from different groups).

In addition, we tested whether the administration of Andro could affect the phosphorylation of ERK in the DRG of the gp120+ddC group. The IOD ratio of p-ERK to ERK in the gp120+ddC+andro group was significantly lower than that in the gp120+ddC group (*p* < 0.01, *n* = 8 for each group) (**Figures 5A,C**). These results suggest that Andro decreased hyperalgesia in the gp120+ddC-treated rats, which was involved in decreasing the phosphorylation and activation of ERK in the DRG of the gp120+ddC-treated rats.

### Molecular Docking of Andrographolide on a Rat P2X7 (rP2X7) Receptor

**FIGURE 6:**
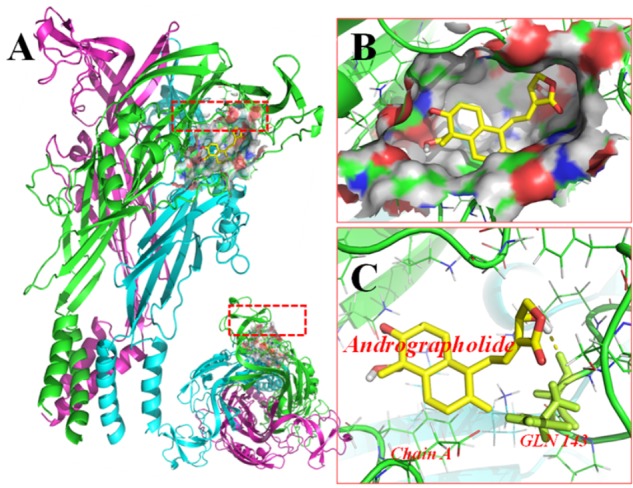
Molecular docking of rP2X7 protein and Andro. Simulation modeling of Andro docking with P2X7 protein was simulated by computer. **(A)** Front and aerial view showed that the best docking position between Andro and rP2X7. The docking position was in the outside of the cell membrane and at the waist of the protein structure, which is near the ATP docking pocket. **(B)** Showed that the best docking pocket between Andro and rP2X7. The ligand, Andro represented in yellow, and P2X7 protein’s active binding pocket displayed in a variety of colors. **(C)** Indicates a possible hydrogen bond formed between the connection residues (GLN143) and ligand. The data showed that Andro could interact with the P2X7 protein.

Molecular docking of Andro on the rP2X7 protein was generated by the AutoDock 4.2. Docking score of rP2X7 and Andro (-7.6 Kcal/mol) showed that Andro enabled the precise fit to interact with the P2X7 receptor (**Table 1**). This precise match enabled Andro to interact with residues near the ATP-binding site (**Figure 6**).

**Table 1:** MOE score of P2X7 protein and andrographolide (kcal/mol).

Mode/Rank	Affinity (kcal/mol)	Dist from rmsb^∗^ l.b	Best mode rmse u.b
1	-7.6	0	0
2	-7.6	38.029	40.189
3	-7.1	33.878	36.206
4	-7.1	24.884	27.297
5	-7	33.828	37.458
6	-6.8	31.025	33.117
7	-6.8	46.705	49.503
8	-6.8	43.364	45.928

*The predicted binding affinity is in kcal/mol (Energy). ^∗^rmsd: RMSD values are calculated relative to the best mode and use only movable heavy atoms. Two variants of RMSD metrics are provided, rmsd/lb (RMSD lower bound) and rmsd/ub (RMSD upper bound), differing in how the atoms are matched in the distance calculation.*

## Discussion

HIV-1 gp120 is a pathogenically relevant factor of HIV-associated neuropathic pain (Zheng et al., 2011; Hao, 2013; Yuan et al., 2014). The neurotoxic effects of anti-retroviral drugs are synergistic for the development of painful neuropathies (Wallace et al., 2007; Zheng et al., 2014). The PWT and PWL in the gp120 and ddC-treated model were much lower than those of the sham group, which was consistent with previous reports (Wallace et al., 2007; Zheng et al., 2014). The P2X7 receptor was involved in inflammatory pain and neuropathic pain (Sperlagh et al., 2006; Skaper et al., 2010; Burnstock, 2013; Mehta et al., 2014; Yang et al., 2016). Our previous study showed that the P2X receptors in the DRG are involved in the transmission of neuropathic pain (Gao et al., 2008; Xu et al., 2012; Liu et al., 2016; Wu et al., 2017; Xie et al., 2017). The present study also showed that in the L4–L6 DRGs, the expression levels of P2X7 mRNA and protein in the HIV-1 gp120 and ddC-treated group were significantly higher than those in the sham group. Upregulated P2X7 receptor in the DRG in the gp120+ddC group was associated with mechanical hyperalgesia and thermal hyperalgesia.

After treatment with Andro in the gp120+ddC group, the upregulation of the P2X7 receptor in the DRG was decreased and mechanical and thermal hyperalgesia was relieved. The data indicated that Andro relieved the hyperalgesia in gp120+ddC treatment rats through down-regulating the elevated expression of the P2X7 receptor. In addition, there was no significant deference for PWT and PWL between the gp120+ddC+andro group and gp120+ddC+andro+A438079 group. The PWT and PWL in gp120+ddC+andro+A438079 group were the same as those in gp120+ddC+A438079 group. The results suggest that in the presence of P2X7 receptor antagonist, Andro no longer shows higher analgesic effects. Thus, the analgesic effects of Andro also have reduction of P2X7 receptor activity.

The upregulation of GFAP indicates the activation of SCGs in DRG (Hanani, 2005; Gu et al., 2010; Suadicani et al., 2010; Ng et al., 2011; Costa and Moreira Neto, 2015). P2X7 receptor expression is expressed in satellite glia cells (Burnstock, 2013, 2014; Kobayashi et al., 2013; Puchalowicz et al., 2014; Gu et al., 2010; Suadicani et al., 2010; Ng et al., 2011). Our experiments showed that the co-expression of GFAP in DRG SCGs after the gp120+ddC-treated rats was increased by double-labeling immunofluorescence. The data indicated the activation of SGCs after nerve injury. Andro decreased the co-expression values of P2X7 and GFAP in the gp120+ddC group. Andro may decrease the co-expression of GFAP and P2X7 in DRG SCGs and reduce the activation of SCGs in DRG. The number of neurons surrounded with GFAP- and P2X7-positive SGCs in the DRG after the gp120+ddC-treated rats was increased and decreased by Andro in the gp120+ddC-treated rats. The activation of SGCs in the DRG is involved in the initiation and maintenance of neuropathic pain. Andro may reduce the activation of SGCs to diminish the expression of the P2X7 receptor and the hyperalgesia in gp120+ddC-treated rats.

The activation of SCGs in DRG promotes the abnormally high production of pro-inflammatory cytokines (Dublin and Hanani, 2007; Clark and Malcangio, 2012). Cytokines, such as IL-1β and TNF-α, can activate the SGCs in the DRG and maintain chronic neuropathic pain. There exist communication between the SGCs and the neurons (Hanani, 2005; Costa and Moreira Neto, 2015). TNF-α can activate TNF-α-R in the DRG neurons, thus the neurons were abnormally excited. NeuN is the specific mark of neurons, its up-regulation means the abnormal changes of neurons. Thus, the intensity of NeuN immunostaining in gp120+ddc group was much higher than that in the sham group. Our results showed that the treatment of gp120 and ddC increased the expression of IL-1β, TNF-α R protein and decreased the expression of IL-10. IL-10 is an anti-inflammatory molecule (Sawada et al., 1999; Pahan et al., 2000; Zheng et al., 2014). The present study revealed that Andro decreased the expression of IL-1β and TNF-α R, as well as increased the expression of IL-10 in the gp120 and ddC-treated rats. Andro may decrease the activation of SGCs in DRG and subsequently reduce the production of pro-inflammatory cytokines and enhance the production of anti-inflammatory cytokines to attenuate the hyperalgesia. Because Andro can be metabolized *in vivo*, Andro may produce anti-inflammatory effects in synergy with its derivatives.

The ATP released from the damaged cells can promote the phosphorylation of ERK (Seino et al., 2006). The activation of the ERK pathway participates in the sensitized primary afferents in the transmission of neuropathic pain (Ji et al., 2009; Lai et al., 2011). Our data showed that the IOD ratio of p-ERK1/2 to ERK1/2 in the gp120 plus ddC group was higher than that in the sham group. The IOD ratios of p-ERK1/2 to ERK1/2 in the gp120+ddC+andro-treated rats were significantly decreased compared to the gp120+ddC group. Thus, Andro inhibited the expression of P2X7 in gp120- and ddC-treated rats, and subsequently reduced the ERK activation in DRG to decrease mechanical and thermal hypersensitivity.

## Conclusion

The present study showed that peripheral nerve exposure to HIV gp120 plus ddC increased mechanical and thermal hyperalgesia in the gp120-plus ddC-treated model rats. The gp120 protein plus ddC increased the expression of the P2X7 receptor in DRG SGCs. The upregulation of the P2X7 receptor in DRG SGCs further promoted the release of pro-inflammatory cytokines (IL-1β and TNF-α) and inhibited the release of an anti-inflammatory cytokine (IL-10). Andro decreased the upregulation and activation of the P2X7 receptor and GFAP. Inhibition of the P2X7 receptor in DRG SGCs reduced the release of pro-inflammatory cytokines, increased the release of anti-inflammatory cytokines, and decreased the phosphorylation of ERK1/2 in the DRG of the gp120-plus ddC-treated rats. Therefore, Andro treatment relieved the mechanical and thermal hyperalgesia in gp120 plus ddC-treated rats.

## Author Contributions

ZY, SO, CZ, LX, ZF, HY, JY, LZ, TJ, SZ, LL, LS, YG, GL, SLiu, HX, CX, and CZ performed the experiments and analyzed the data. ZY wrote the paper. SLia designed the experiments, wrote, and revised the paper.

## Conflict of Interest Statement

The authors declare that the research was conducted in the absence of any commercial or financial relationships that could be construed as a potential conflict of interest.

## Footnotes

1 http://www.who.int/gho/hiv/en/

2 https://swissmodel.expasy.org/

3 http://www.pymol.org/
